# Light‐Induced Orthogonal Reactivity of Photoinitiators from One‐Electron Reduction to Nanocomposites

**DOI:** 10.1002/anie.202512534

**Published:** 2025-12-21

**Authors:** Max Schmallegger, Mathias Wiech, Georg Gescheidt

**Affiliations:** ^1^ Institut für Physikalische und Theoretische Chemie Technische Universität Graz Stremayrgasse 9 Graz A–8010 Austria

**Keywords:** Nanocomposites, Orthogonal reactivity, Photoinitiators, Plasmonic nanoparticles, Redox reactions

## Abstract

Photoinitiators for radical polymerization have been well established. This minireview indicates a palette of applications beyond their original scope. Depending on the experimental conditions, the primary radicals generated upon photolysis act as reducing agents for metal salts. This opens new possibilities for using photoinitiators as versatile and convenient tools for the preparation of metal/polymer nanocomposites.

## Graphical abstract

Irradiating a mixture of a photoinitiator, a metal salt, and a monomer in an alcoholic solvent or aqueous solution provides a plasmonic polymer‐metal nanocomposite in a highly convenient way.

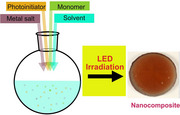

## Introduction

Metal nanoparticles have gained importance in fields reaching from biomedical applications to catalysis. Producing plasmonic particles displaying a reasonable size and stability (no agglomeration) has been a particular challenge in this field. Here several approaches have been developed, but they mostly involve complex and costly procedures. In 2006, Scaiano and coworkers^[^ ^1^ ^]^ have shown that photolyzing an *α*‐hydroxyketone photoinitiator in the presence of an Au(III) salt leads to the photo‐induced formation of unprotected Au nanoparticles. This principle has been carried further, *e*.*g*. for the synthesis of Cu(I) from Cu(II),^[^ ^2^ ^]^Ag,^[^ ^3^, ^4^, ^5^ ^]^ or Pd^[^ ^6^, ^7^ ^]^nanoparticles. Moreover, polymer‐bound *α*‐hydroxyketones were utilized for immobilizing the metal particles.^[^ ^8^ ^]^ Bisacylphosphane oxide initiators (BAPOs) have been reported as photoactive electron transfer agents. In the presence of Cu(II) salts Cu(I) was generated upon photolysis of bis(mesitoyl)phosphane oxide, which then promoted controlled radical polymerizations or click reactions.^[^ ^2^, ^9^, ^10^, ^11^, ^12^, ^13^, ^14^, ^15^ ^]^ Under aqueous/alcoholic conditions BAPOs were shown to reduce Cu(II) to elemental Cu. Analogous reactions have been reported for benzoin derivatives.^[^ ^16^, ^17^ ^]^ This minireview aims to clarify the mechanism behind the one‐electron reducing capabilities of photoinitiators and based on these insights, to outline their scope of applications.

## Redox Properties of Photoinitiators

**Scheme 1:**
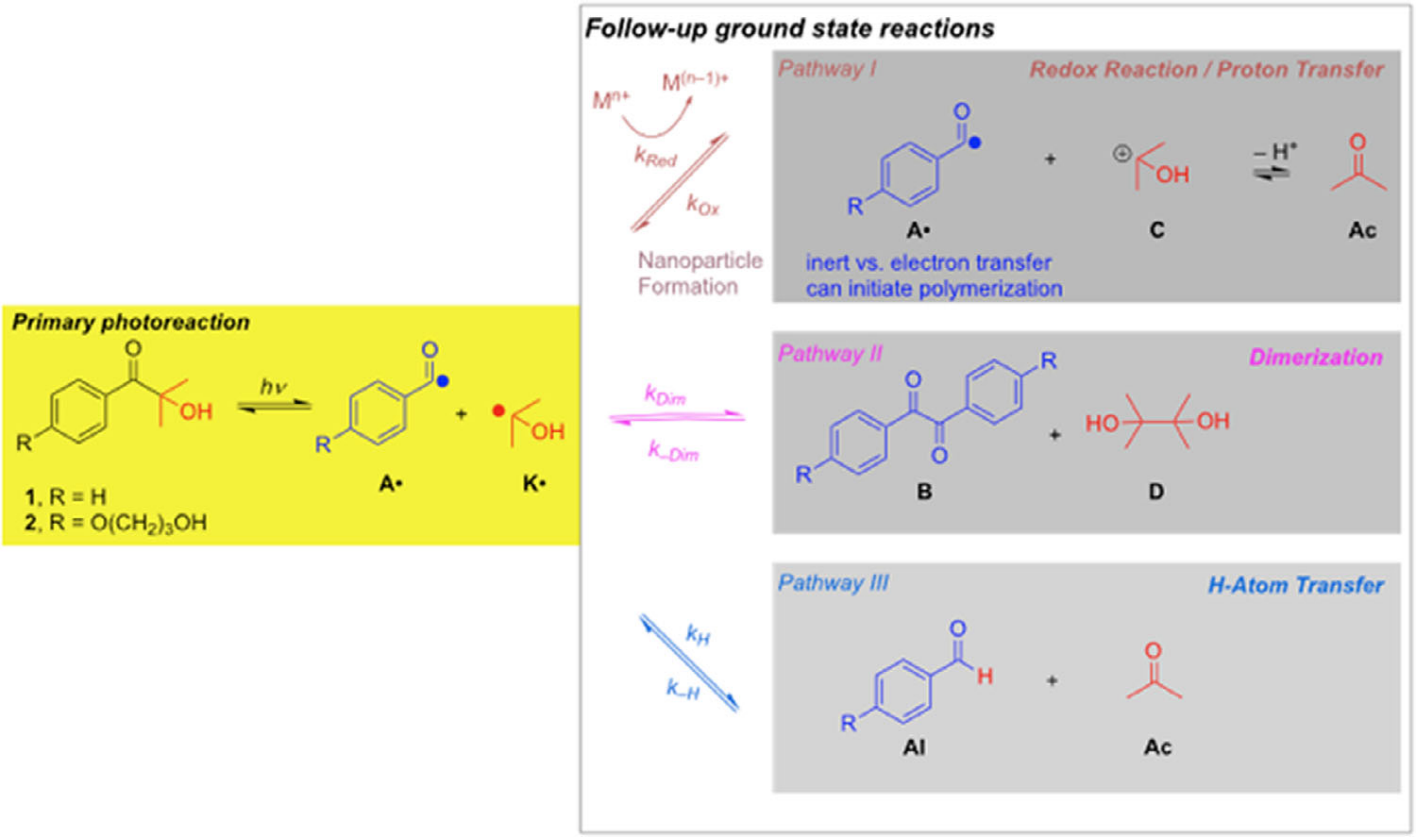
Photoinduced reaction of *α*‐hydroxyketones **1** and **2,** followed by the ground‐state reaction pathways of the primary radical pair **A**•/**K**•.

Upon irradiation, *α*‐hydroxyketones, *e*.*g*., (1‐[4‐(2‐hydroxyethoxy)phenyl]‐2‐hydroxy‐2‐methyl‐1‐propane‐1‐one (**1**) or 2‐hydroxy‐2‐methylpropiophenone (**2**)  undergo *α*‐cleavage yielding the corresponding aroyl (**A**•) and ketyl (2‐hydroxy‐2‐propyl) radicals (**K**•, Scheme 1). It was shown that both radicals initiate radical polymerization and their reactivity in these terms has been well explored.^[^ ^18^, ^19^, ^20^, ^21^, ^22^, ^23^, ^24^, ^25^, ^26^ ^]^

Moreover, *α*‐hydroxyketones have been reported as phototriggered sources for one‐electron reductions of various metal salts.^[^ ^1^, ^3^, ^4^, ^5^, ^6^, ^7^, ^8^, ^27^ ^]^ In a similar way *α*‐aminoketones have been shown to be redox active.^[^ ^3^, ^28^ ^]^Here we concentrate on *α*‐hydroxyketones as reducing agents. It is essential to take a closer look at the follow‐up reactions based on the initial photogenerated (via excitation, intersystem crossing and *α*‐cleavage in the triplet state) radical pair **A**•/**K**• (Scheme 1). With of monomers both radicals initiate polymerizations.^[^ ^29^, ^30^ ^]^ In the presence of reducible metal salts, M^n+^, the 2‐hydroxy‐2‐propyl radical (**K**•) acts as reductant (–0.61 V versus SCE (saturated calomel electrode), in MeCN^[^ ^31^, ^32^, ^33^ ^]^) yielding M^(n–1)+^ and cation **C**. This latter highly reactive cation^[^ ^28^, ^34^ ^]^ converts to acetone (**Ac**, upon deprotonation, Scheme 1, *Pathway I*).^[^ ^28^ ^]^ *Pathway II* in Scheme 1 indicates recombination reactions of **A**• and **K**• producing dimers **B** (benzil‐type) and **D** (butane‐2,3‐diol, pinacol). *Pathway III* comprises hydrogen‐atom transfer from **K**• toward **A** yielding aldehyde **Al** and acetone (**Ac**) established by NMR and CIDNP.^[^ ^28^, ^35^, ^36^, ^37^ ^]^ It is noteworthy, that **A**• type radicals are bad hydrogen abstractors. Hence the formation of the aldehyde **Al** is bound to the transfer of hydrogens in *β*‐positions of radical centers (e.g., **K**• or growing polymer chains; such hydrogens possess favorable bond dissociation energies^[^ ^38^ ^]^). The palette of reactions in Scheme 1 presents coupled competitive reactions, which are reversible. Accordingly, their efficiency is ruled by kinetics. Photolysis of **2** in MeCN yields **Al**, **Ac**, **B**, and **D** (via *Pathways II* and *III*, Scheme 1, see Supporting Information for the corresponding NMR/EPR spectra). Notably, in the presence of PdOAc_2_ **Al** and **D** are not detectable indicating that the electron transfer reaction producing the reduced metal salt and **Ac** (by proton‐coupled electron transfer) is faster than the bimolecular reactions *II* and *III*. Accordingly, *Pathway I* become dominating. An important finding is that **A•** does not participate in the redox reaction. Instead, **A**• dimerizes leading to 1,2‐dione **B**. Whereas **B** cannot be unequivocally identified by NMR (many overlapping lines in the aromatic region), irradiation of the reaction mixture upon addition of an electron donor (e.g., triethylamine) produces the EPR spectrum of **B** ^•–^ clearly underlining the formation of **B** (see also below).

**Scheme 2:**
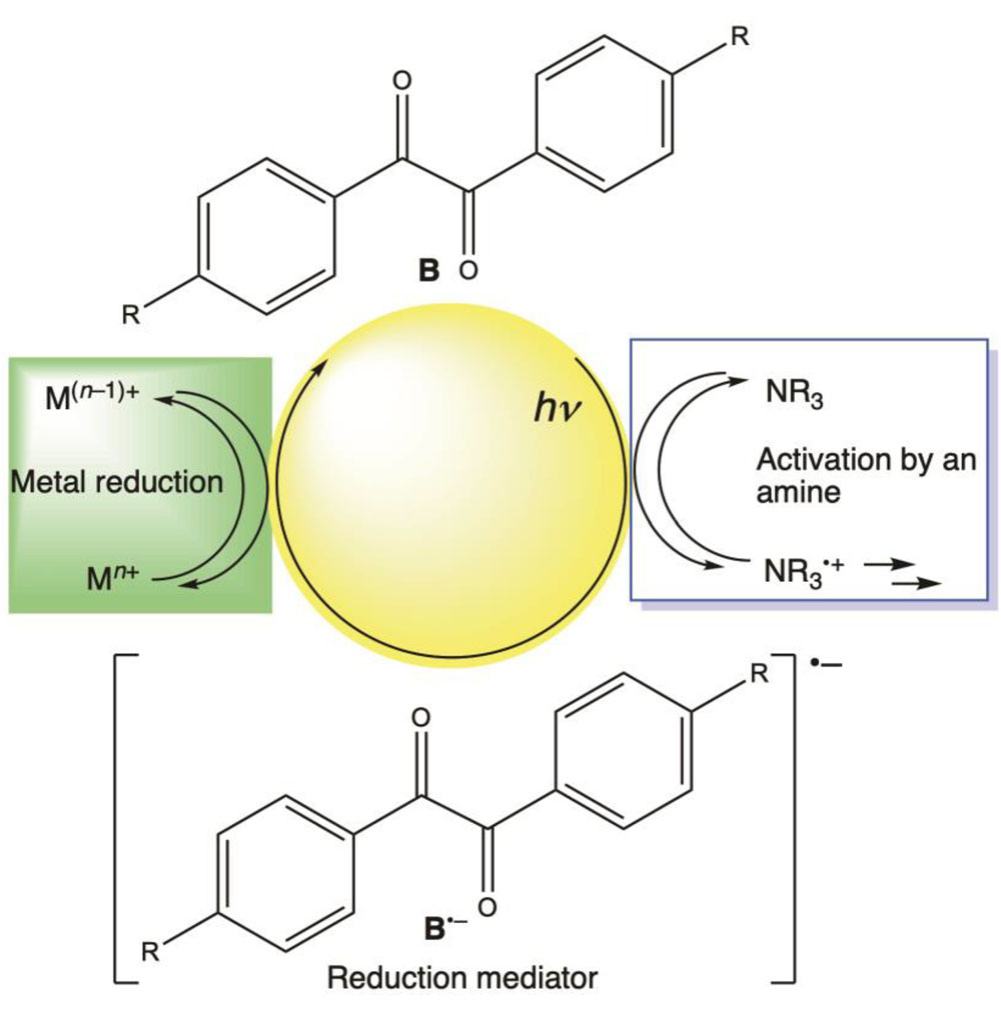
The photoinduced reaction between benzil (B) and an amine producing B^•–^ as an electron‐transfer mediator (photoreduction of B).

*Pathway II* rationalizes the observation that *α*‐hydroxyketones utilized at high concentrations act as reducing agents particularly in the presence of amines (impurity in N,N‐Dimethylformamide (DMF)).^[^ ^8^ ^]^ Although it has provisionally been suggested that NHMe_2_ may add to the aroyl radical forming an additional reducing agent it is more likely that **B** is the decisive agent: Photolysis of **2** with triethylamine added to the reaction solution produces radical anion **B** ^•–^, which serves as an electron‐donating species during irradiation (see Supporting Information).^[^ ^36^ ^]^ Thus a “pseudo catalytic” cycle emerges, in which the photoinduced electron transfer between an amine and a (substituted) benzil yields an amino radical cation and the benzil radical anion, the latter acting as a reducing agent (Scheme 2).^[^ ^36^ ^]^

Ketyl‐type radical producing photoinitiators have been utilized for the synthesis of Au, Ag, Cu, Co, Pt, and Ru nanoparticles^[^ ^3^, ^28^ ^]^with average sizes between *ca*. 5 and 40 nm. These depend mainly on kinetics (this will be discussed in the final part of this review).

A delicate balance between the reaction channels displayed in Schemes 1 and 2 is the basis underlying the photoredox activity of *α*‐hydroxyketones. Whereas the photo‐induced *α*‐cleavage of a ketone (Norrish type I reaction) is the source of the stochiometric ground‐state redox agent **K•**, Scheme 2 indicates an additional reaction channel, which relies on the radical anion **B^•–^** originating in *Pathway II* acting as a reduction mediator under continuous irradiation. Experimentally this is established by EPR spectroscopy (see Supporting Information).^[^ ^36^ ^]^

**Scheme 3:**
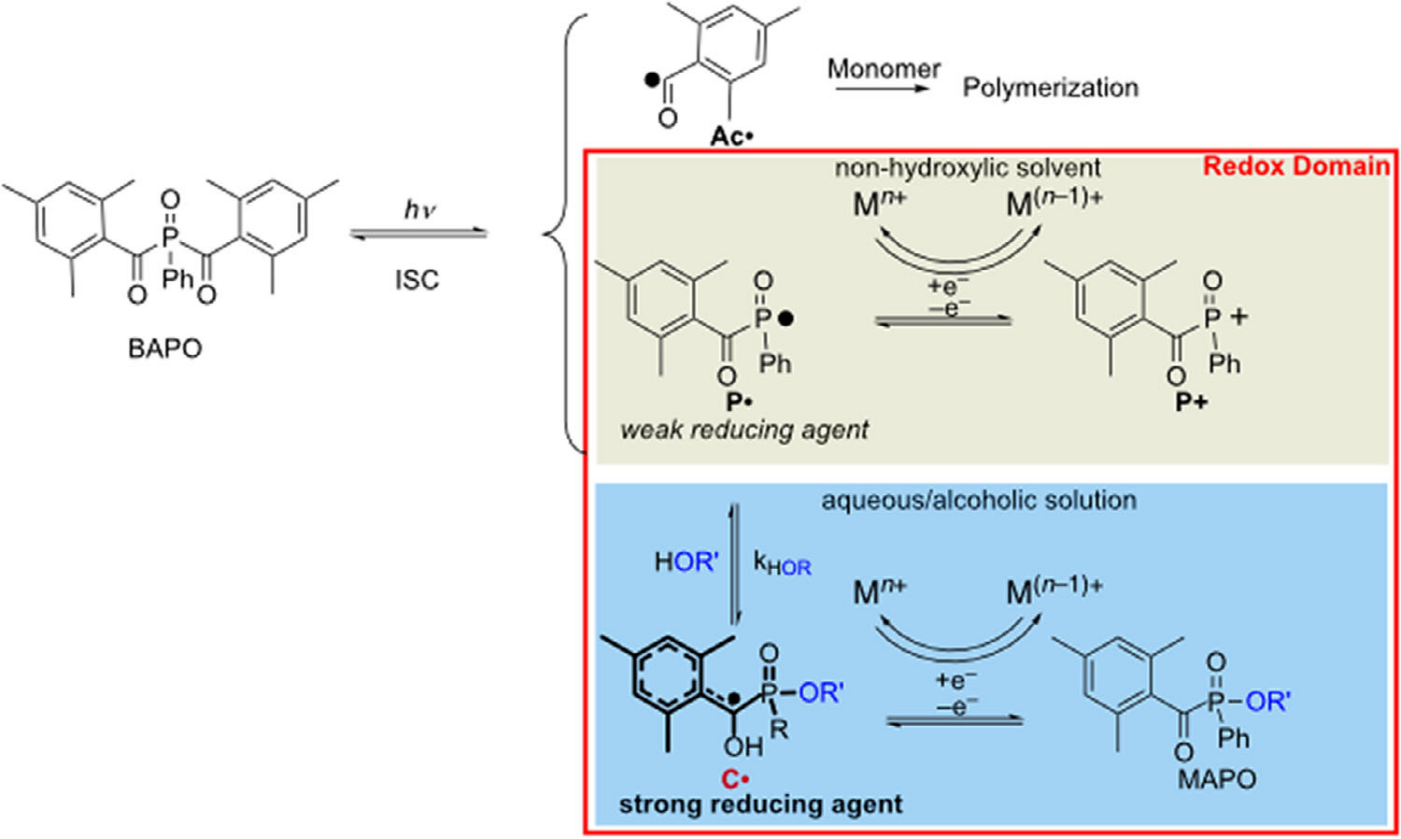
Photolysis of BAPO and its follow‐up reactions in aqueous and alcoholic solution.

Mono‐ and bisacyl phosphane oxides have been well established as photoinitiators for radical polymerization.^[^ ^39^, ^40^, ^41^, ^42^, ^43^ ^]^ It has been shown that these molecules are also able to reduce Cu(II) to Cu(I)^[^ ^13^, ^44^ ^]^ with rate constants of *ca*. 2 x 10^9^ M^−1^s^−1^. Here, the phosphonyl radical **P**• formed from BAPO (bis(2,4,6‐trimethylbenzoyl)phenylphosphane oxide) acts as the reducing agent (being converted to the phosphonyl cation **P**+ (Scheme 3).^[^ ^7^ ^]^ Analogous reactions have been applied for triggering Cu(I)‐induced polymerizations.^[^ ^15^, ^45^ ^]^

Notably, in aqueous and alcoholic solvents BAPO and related bisacylphosphanes act as even more efficient reducing agents.^[^ ^46^ ^]^ This can be attributed to the formation of **C•** by a nucleophilic attack of RO^–^ on the phosphorus center followed by tautomerization (with H^+^ as the counterion, Scheme 3). Radical **C•** is of benzoyl‐type and by carrying electron‐rich substituents it acts as an electron donor.^[^ ^37^, ^47^ ^]^ Owing to its delocalized character, **C•** does not add to double bonds and therefore, cannot initiate radical polymerizations. However, upon its oxidation **C•** converts to a monoacylphosphane oxide (MAPO), which, again is a photoinitiator. Upon photolysis of MAPO (at sufficiently short wavelengths < 430 nm),^[^ ^37^ ^]^ the corresponding radical of type **A•** (see Scheme 1) can initiate radical polymerizations. The corresponding phosphorus‐centered radical efficiently adds to double bonds and can act as a (weak) reducing agent (upon formation of a phosphorus‐centered cation).

It has been reported that photolysis of a bisacylgermane in the presence of diphenyliodonium hexafluorophosphate initiates a cationic polymerization in analogy to BAPOs and MAPOs.^[^ ^9^ ^]^ The authors have shown that this reaction relies on the formation of radicals. Tentatively, they suppose that the initially formed germyl radical is converted (oxidized) to a germyl cation, which, then initiates the polymerization.^[^ ^48^ ^]^

**Scheme 4:**
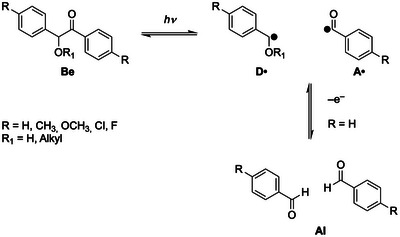
Photolysis of Benzoin (**Be**) derivatives and tentative mechanism for the oxidation of benzoin **Be**.

A highly related mechanism holds for the photolysis of benzoin (**Be**) derivatives. Here photolysis produces a benzoyl (acyl) radical and a benzyl radical (**D**•) (Scheme 4).^[^ ^16^, ^49^, ^50^ ^]^ It has been shown that benzoin acts as a reducing agent for Cu(II).^[^ ^17^ ^]^ The mechanism is not unequivocally established. However, the observation, that only benzoin derivatives with R═H (Scheme 4) are redox active implies, that the primary *α*‐hydoxybenzyl radical (**D**•) has to undergo hydrogen‐atom transfer to act as a reducing agent and form the corresponding aldehyde.^[^ ^17^ ^]^ The above examples indicate that ketyl, phosphane‐oxide, germanium‐based, and electron rich benzyl radicals may serve as electron‐transfer active species.

## Orthogonal Initiation and Reduction

**Scheme 5:**
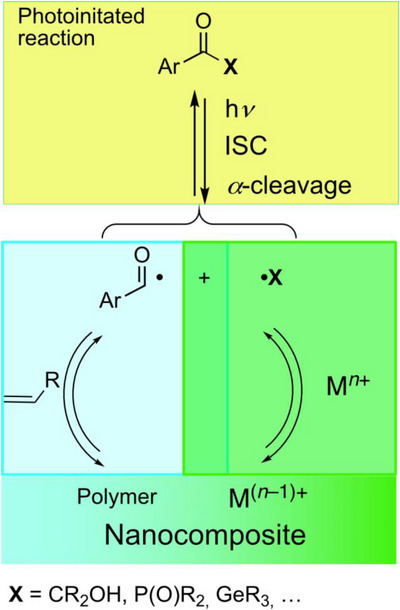
Simplified representation of the orthogonal redox and polymerization reaction of selected photoinitiators and a sketch for the use of this reactions to produce nanocomposites.

Based on the primary radical pairs (**A**•/**K**• or **P**•/**Ac**•) only **K**• and **P**• (and **C**•**/D**•) are redox active. The aroyl radicals (**A**• or **Ac**•) do not act as reducing agents. Accordingly, these initiators are able to act as orthogonal reagents: whereas the aroyl radicals initiate radical polymerization, their counterparts are suitable one‐electron reductants (Scheme 5).

This opens the door of simultaneously starting a redox reaction (acting as a reducing agent) and a radical polymerization (orthogonality) upon irradiation. The species (*e*.*g*., a metal atom) formed by the redox reaction is immediately immobilized/stabilized within a polymer matrix.

When utilizing this orthogonal reactivity, it is important to consider the reversible character of each reaction step. Thus, kinetics rule the outcome of these reactions. Except for ketyl radicals, the reduction potentials of the P (or Ge) centered radicals have not yet been determined (our attempts to establish these values by photo‐modulated cyclovoltammetry unfortunately failed) but particularly radicals of type **C•** are sufficient to reduce Cu(II) to elemental Cu. The rate constants of the reactions behind the formation of polymers and the redox processes have been established.^[^ ^18^, ^19^, ^29^, ^30^, ^39^, ^44^, ^47^, ^51^, ^52^ ^]^ When developing simultaneous redox/polymerization reactions, a careful choice of reactant concentrations is crucial. The starting reaction is photoinduced, therefore, in addition to the quantum efficiency of the initial *α‐*cleavage, the penetration depth into the reaction solution at the applied wavelength, and the light intensity determine the concentration of the redox‐active species. With the knowledge of this concentration, it is possible to adjust the rates for nanoparticle formation. Kinetic simulations thus serve as a tool to predict the size of the nanoparticles. Using established rate constants, we have constructed a modelling tool, which should facilitate the planning of these reaction sequences (based on COPASI,^[^ ^53^ ^]^ see Supporting Information).

**Figure 1:**
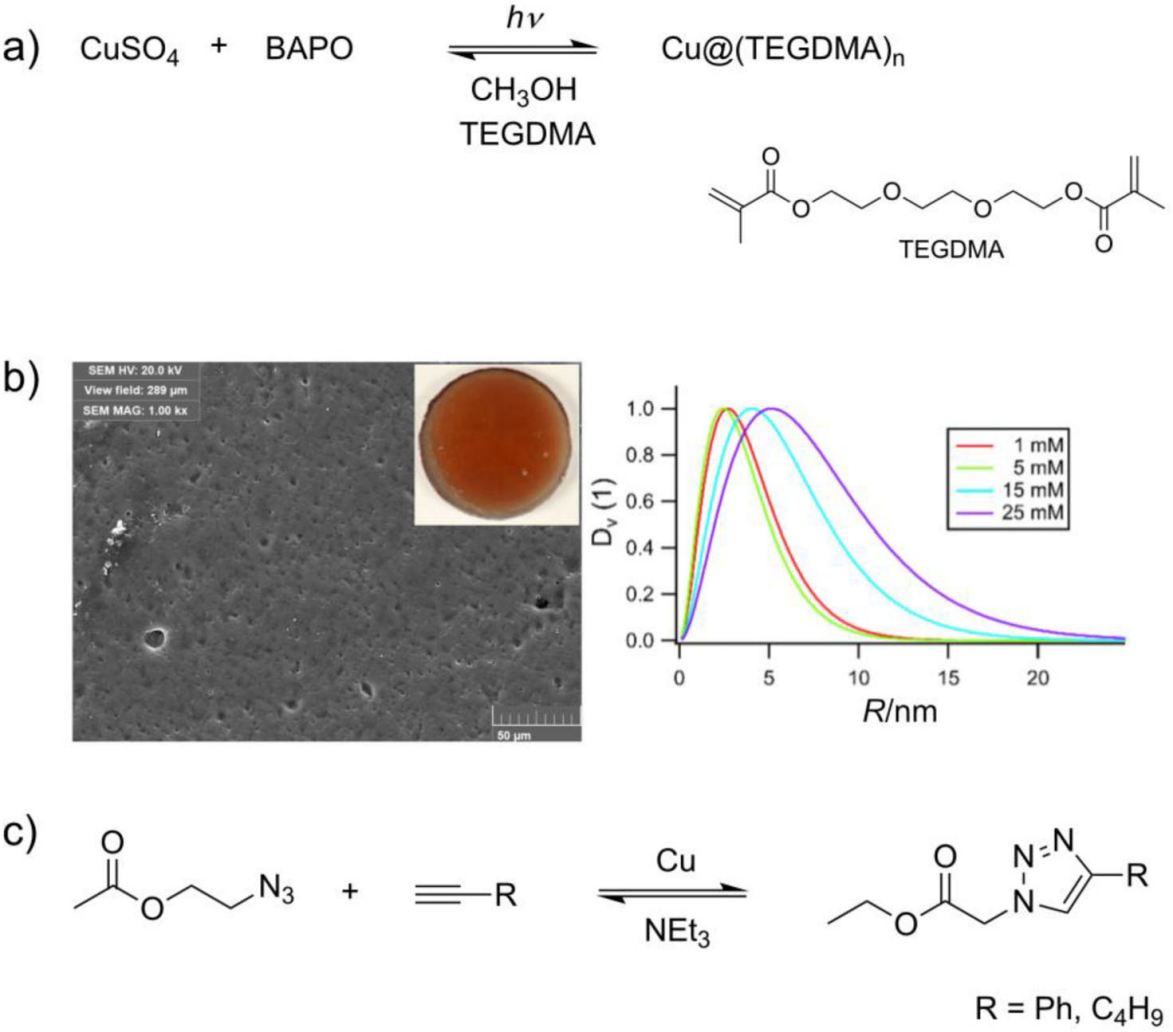
a) Preparation of Cu@TEGDMA; b) electron micrograph of Cu@TEGDMA (the insert shows the macroscopic appearance with the typical red color for Cu plasmon absorption) and the size distribution of Cu nanoparticles determined by SAXS; c) Cu‐catalyzed click reaction (adapted from ^[^ ^54^ ^]^).

Performing photolyses of BAPO in the presence of metal salts and suitable monomers produces metal‐polymer composites. Figure 1 shows the composite formed upon photolyzing a mixture of BAPO, CuSO_4_ and triethylene glycol dimethacrylate (TEGDMA) in methanol. Such formulations produce particle sizes between 2 and 7 nm with relatively broad distributions established by small‐angle X‐ray scattering (SAXS), scanning electron micropscopy (SEM), and X‐ray scattering.^[^ ^54^ ^]^

We have evaluated these composites as catalysts for a Cu‐catalyzed alkyne‐azide cycloaddition.^[^ ^54^ ^]^ Yields vary between 97% and 85% (compatible with established data).^[^ ^55^, ^56^, ^57^, ^58^ ^]^ When the nanocomposites are re‐used for additional runs, their catalytic efficiency decreases gradually – mainly caused by air oxidation of Cu to Cu(II).

The ability of *α*‐hydroxy/*α*‐aminoketones and bisacylphosphane oxides to act as electron‐donating reagents upon photolysis has been discovered several years ago. However, more recent, in‐depth mechanistic investigations have shown that the initial radical pairs created upon irradiation may act as sources for two orthogonal domains of reactivity. Whereas *α*‐hydroxy/*α*‐amino based radicals (**K**•‐type) have straightforwardly been identified as redox active species, less focus has been set to their acyl‐type counterparts **A**•. These latter radicals are not electron transfer agents. However, in the presence of monomers, they initiate radical polymerizations (well‐established). Only dimers of type **B** serve as electron transfer mediators in the presence of bases (Schemes 1 and 2). In the same way, **Ac**• is generated from BAPOs. Here, the electron transfer‐active species is either phosphonyl radical **P**• or the substantially more efficient benzoyl‐type radical **C**• emerging from the rapid reaction of **P**• with water or alcohols.

## Conclusion

The common feature in the reactivity patterns of *α*‐hydroxy/*α*‐aminoketones and bisacylphosphane oxides shown in Schemes 1 and 3 is the competitive character of the (equilibrium) reactions connecting photolysis and the follow‐up ground‐state reactions. Accordingly, the kinetics of these generally bimolecular reactions are decisive to control the outcome of the reactions including the growth/sizes of the metal nanoparticles and the protecting polymer matrix. Here, the quantum efficiency of the initial *α*‐cleavage, the choice of substrates, solvents, and the stoichiometric ratios of the reactants must be considered (the predicting tool provided in the Supporting Information should facilitate finding optimum reaction conditions).^[^ ^47^ ^]^ The extended knowledge of mechanistic details is a prerequisite to streamline decisive reaction parameters (irradiation intensity, duration of the irradiation, concentration ratios) to achieve nanocomposites with predictable (and reproducible) properties. Bearing in mind that there is a plethora of strategies to obtain metal nanoparticles with well‐defined sizes (size distributions),^[^ ^8^, ^59^ ^]^ the approach introduced here is rather simple. With the knowledge of rather well available rate constants, the properties of the nanocomposites are predictable. The photoinitiators discussed here are well‐established and commercially available. Salts of Ag^+^, Au^+^, Pd^2+^, Pt^2+^, and Cu^2+^ have been shown as being reducible by radicals emerging from the *α*‐cleavage of photoinitiators. Any monomer capable of undergoing radical polymerization is feasible, with the option of incorporating porogens. The homogeneous nature of the starting solutions guarantees a well‐dispersed distribution of the nanoparticles throughout the polymer matrix.

The spatial and temporal control provided by photoinduced initiation makes this technique particularly suitable for advanced applications including 3D printing or the precise embedding of molecular (organic/inorganic) species in defined oxidation states. Moreover, application of this methodology for plasmonic coatings may prove valuable.^[^ ^60^ ^]^

This mini review aims to highlight the convenience and versatility of photoinitiator‐based orthogonal redox/polymerization systems for the fabrication of diverse nanocomposites. The authors hope that the straightforward toolbox presented here will encourage its adoption across a wide range of metal–polymer nanocomposite applications.

## Conflict of Interests

The authors declare no conflict of interest.

## Supporting information



Supporting Information

Supporting Information

## Data Availability

The data that support the findings of this study are available from the corresponding author upon reasonable request.
