# Supplementary material for: Light‐Induced Orthogonal Reactivity of Photoinitiators from One‐Electron Reduction to Nanocomposites
Source: Angew Chem Int Ed Engl. 2025 Dec 21;65(4):e12534. doi: 10.1002/anie.202512534 (PMC12828448; doi:10.1002/anie.202512534)
Supplement: Supplementary file 1 — Supporting Information [file ANIE-65-e12534-s001.pdf]

SUPPORTING INFORMATION

Light-Induced Orthogonal Reactivity of Photoinitiators from One-Electron Reduction to Nanocomposites

Max Schmallegger, Mathias Wiech, and Georg Gescheidt*

Dr. Max Schmallegger, Mathias Wiech, Prof. Dr. Georg Gescheidt
Institute of Physical and Theoretical Chemistry, TU Graz, Stremayrgasse 9, A–8010 Graz

This information provides supplementary NMR and EPR spectra, as well as details on the kinetic modelling of the redox- and radical-reactions shown in the manuscript. The two corresponding COPASI simulation routines are provided in two separate files.

Contents

1. Spectroscopy 2
   1.1. NMR Spectroscopy 2
   1.2. NMR Reference Spectra 4
   1.3. EPR Spectroscopy 7
2. Kinetic Modelling 8
3. References 10

1. Spectroscopy

2-Hydroxy-4′-(2-hydroxyethoxy)-2-methylpropiophenone (**2**), 2-hydroxy-2-methylpropiophenone (**1**, 97%), Palladium acetate (98%) and triethylamine (99%) were purchased from Sigma Aldrich. All substances were used as received.

1.1. NMR Spectroscopy

NMR spectra were acquired on a Bruker Avance III 400 MHz system in acetonitrile-D3 (99.8% D, Eurisotop GmbH) in conventional NMR tubes. ^{13}Carbon spectra were recorded with proton decoupling. The reported chemical shifts δ are referenced against tetramethylsilane (TMS, ^{1}H: 1.94 ppm, ^{13}C: 1.39 ppm), using the residual non-deuterated solvent as an internal reference. All spectra were processed in MestReNova 15.1.

Samples containing 3.5 mg **2** (20 mM) and/or 3.5 mg $Pd(OAc)_2$ (20 mM) in 700 µl ACN-D3 were prepared directly in 5 mm NMR tubes. Oxygen was removed from the solutions by bubbling with nitrogen for five minutes. The samples were LED-irradiated in a custom-build photoreactor at 365 nm for 5 minutes. NMR spectra were recorded immediately before and after irradiation.

Figure S1 shows ^{1}H NMR spectra of **2** before/after irradiation and a ^{13}C NMR spectrum of the same sample after irradiation. H-atom transfer after photolysis predominantly yields the corresponding aldehyde **Al** and acetone **Ac** (Pathway III, Scheme 1, main text). Both species exhibit distinct singlet ^{1}H resonances at 9.86 ppm (**Al**) and 2.08 ppm (**Ac**). This assignment is supported by ^{13}C NMR. In addition, dimerization of ketyl-type radicals produces pinacol **D** (Pathway II, Scheme 1, main text).

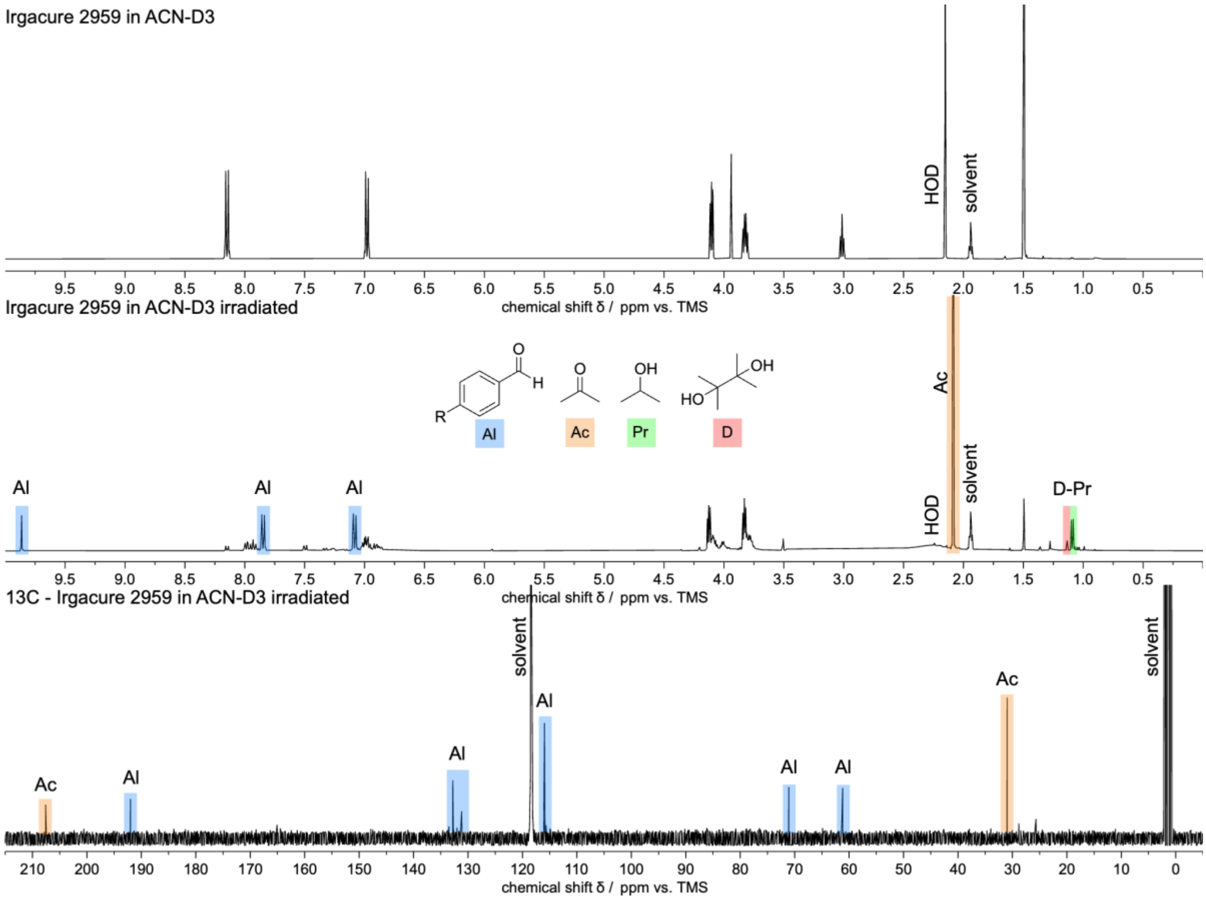

Figure S1. Top/middle: ^{1}H NMR spectrum of **2** in ACN-D3 before/after 365 nm LED-irradiation. Bottom: ^{13}C NMR spectrum acquired after irradiation.

In contrast, ^{1}H and ^{13}C NMR spectra recorded from a solution containing **2** and $Pd(OAc)_2$ after light irradiation show solely acetone as the primary reaction product (Figure S2). A brownish colour of the sample after irradiation indicates the formation of Pd-nanoparticles and thus the predominance of redox-reactivity (Pathway I, Scheme 1, main text).

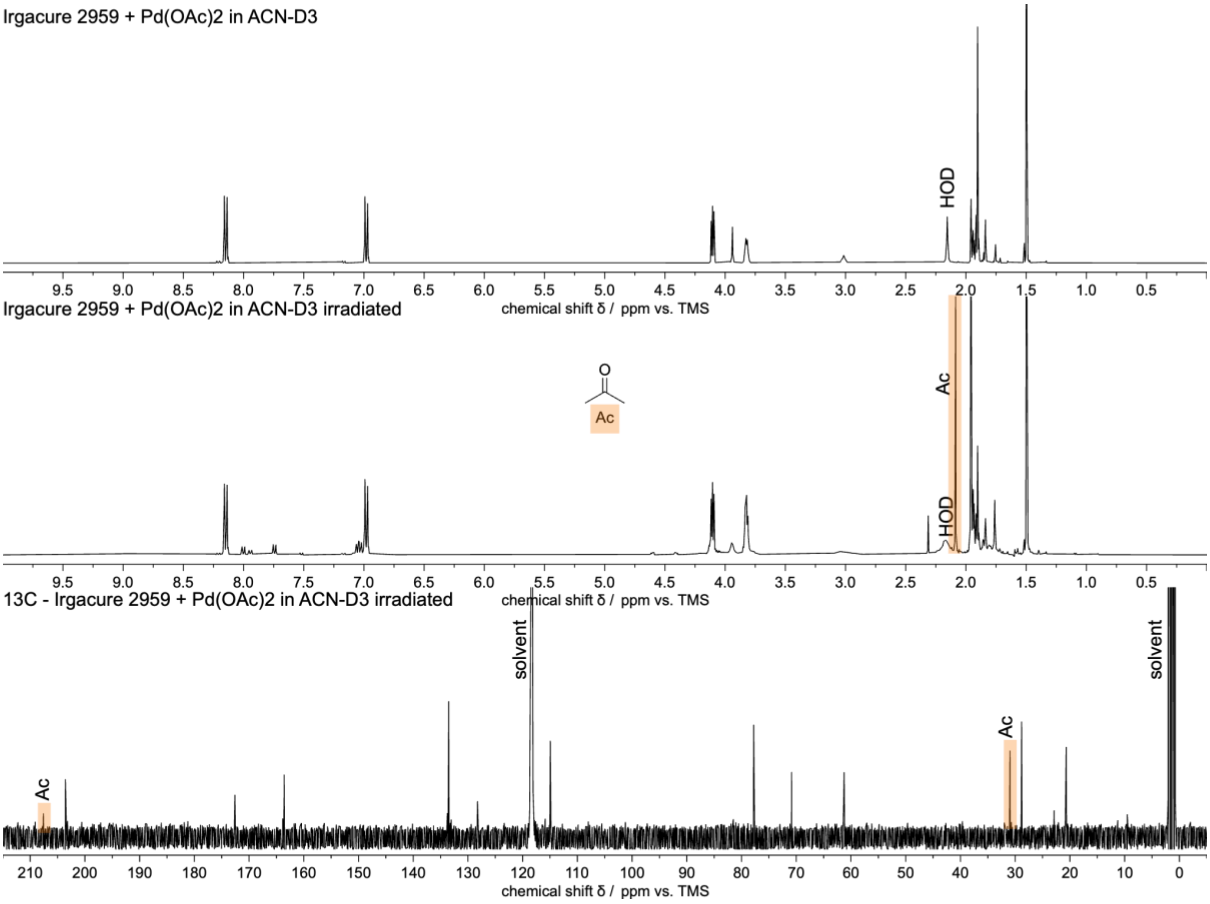

Figure S2. Top/middle: ^{1}H NMR spectrum of a solution containing **2** and $Pd(OAc)_2$ in ACN-D3 before/after 365 nm LED-irradiation. Bottom: ^{13}C NMR spectrum acquired after irradiation.

1.2. NMR Reference Spectra

2-hydroxy-1-[4-(2-hydroxyethoxy)phenyl]-2-methylpropan-1-one (**2**)

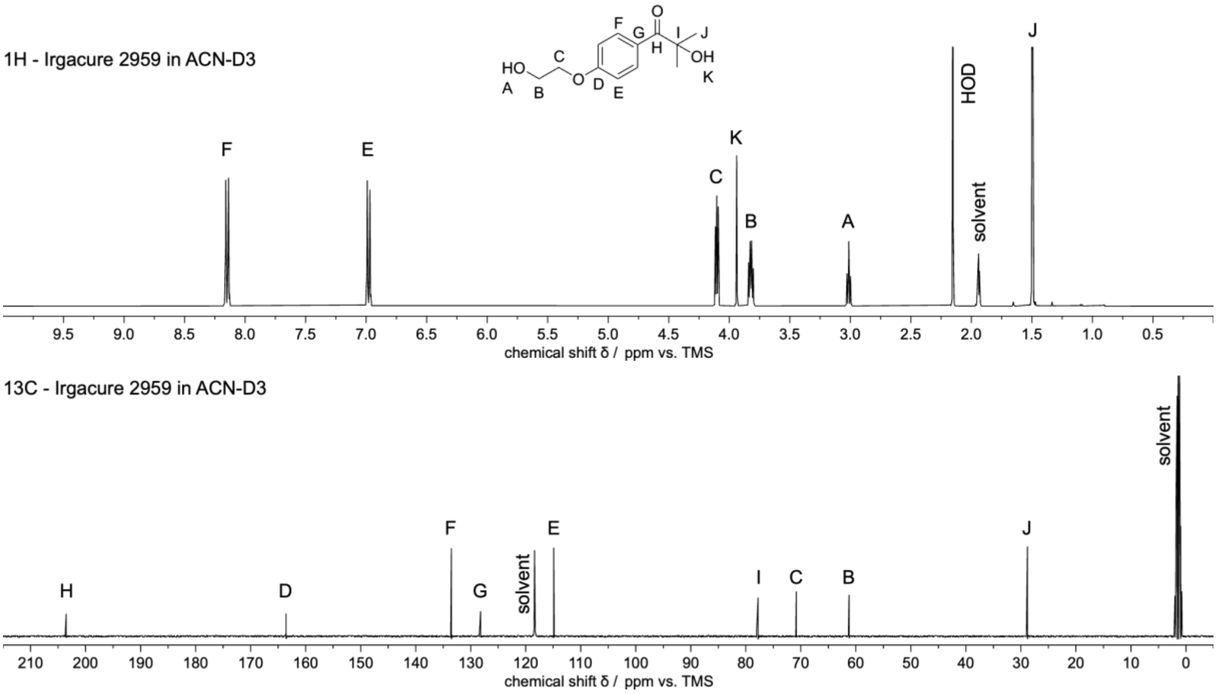

Figure S3. ^{1}H and ^{13}C NMR spectra of **2**.

[^{1}H, δ in ppm vs TMS, 400 MHz]: 8.15 (m, E); 6.98 (m, D); 4.10 (t, C); 3.94 (s, G); 3.82 (dt, B); 3.01 (t, A); 1.50 (s, F)

[^{13}C, δ in ppm vs TMS, 100 MHz]: 203.5 (H); 163.6 (D); 133.5 (F); 128.2 (G); 114.9 (E); 77.8 (I); 70.8 (C); 61.2 (B); 28.8 (J)

Palladium(II)acetate

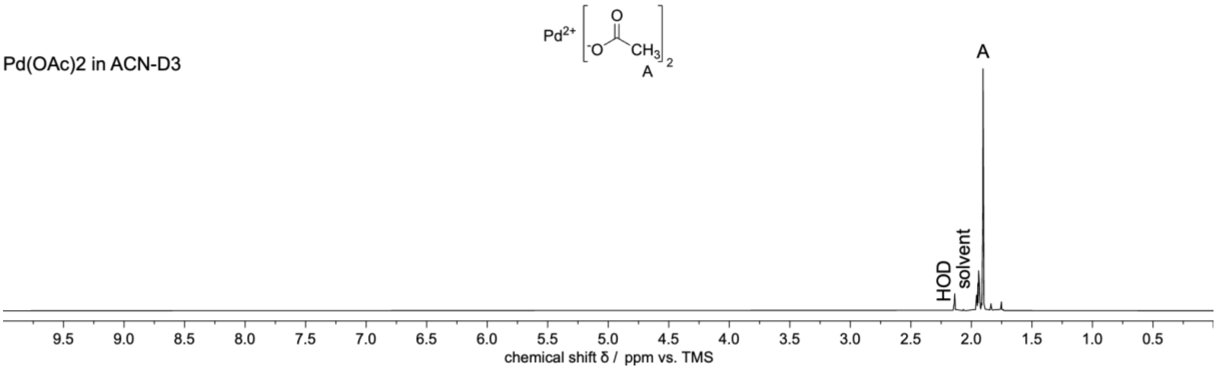

Figure S4. ^{1}H NMR spectrum of $Pd(OAc)_2$.

[^{1}H, δ in ppm vs TMS, 400 MHz]: 1.90 (s, A)

Propan-2-one (Acetone)

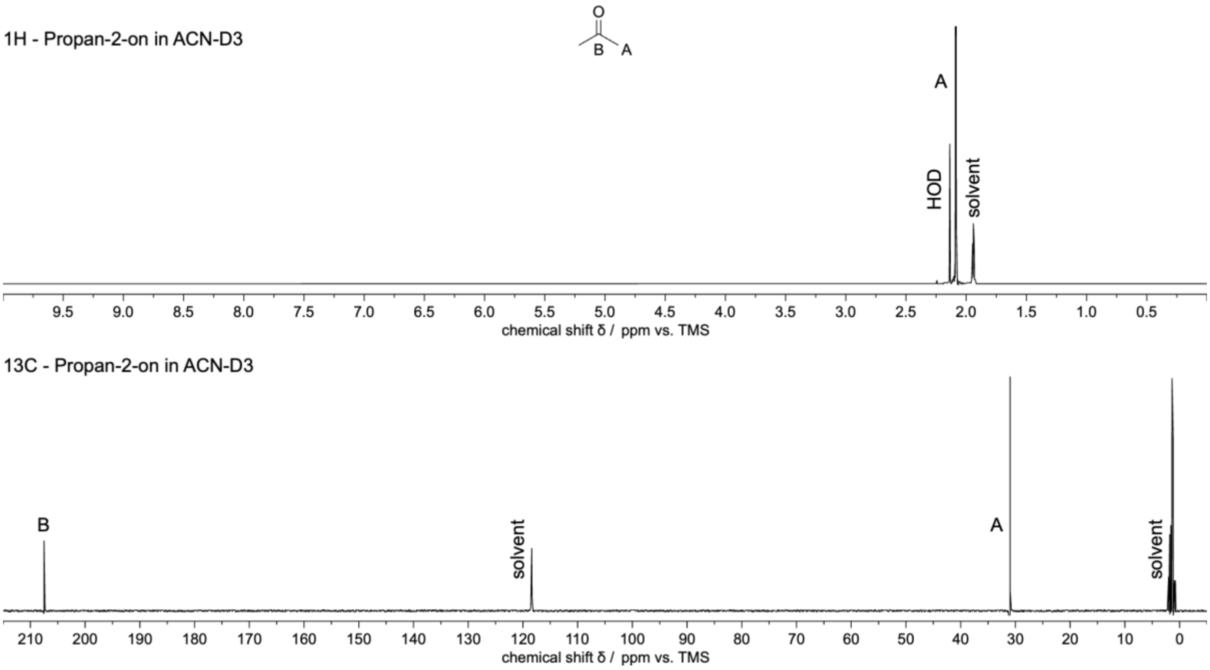

Figure S5. ^{1}H and ^{13}C NMR spectra of acetone.

[^{1}H, δ in ppm vs TMS, 400 MHz]: 2.08 (s, A)

[^{13}C, δ in ppm vs TMS, 100 MHz]: 207.5 (B); 30.9 (A)

Propan-2-ol

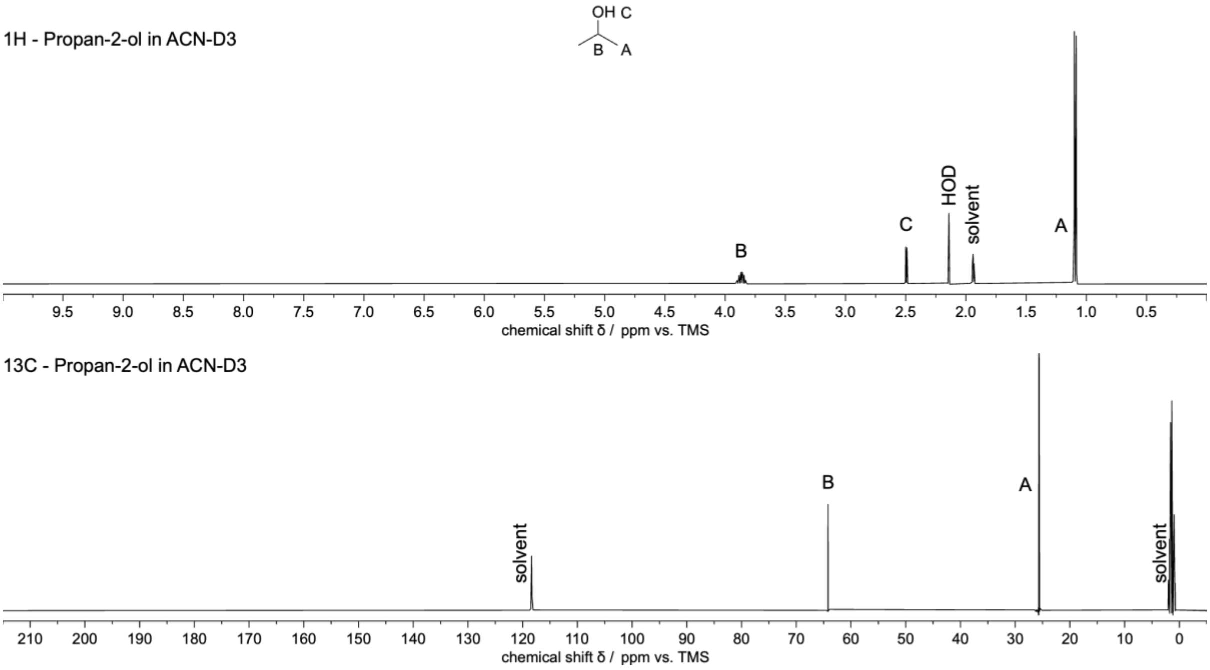

Figure S6. ^{1}H and ^{13}C NMR spectra of 2-propanol.

[^{1}H, δ in ppm vs TMS, 400 MHz]: 3.86 (m, B); 2.49 (d, C); 1.09 (d, A)

[^{13}C, δ in ppm vs TMS, 100 MHz]: 64.2 (B); 25.6 (A)

2,3-Dimethylbutane-2,3-diol (Pinacol)

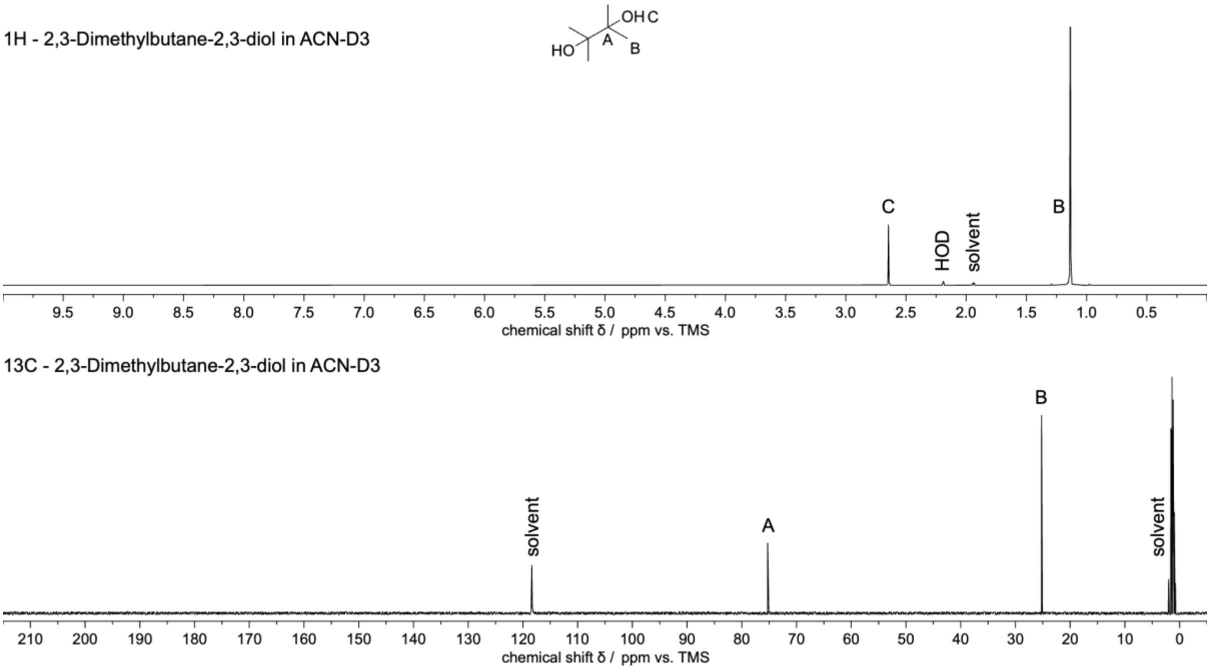

Figure S7. ^{1}H and ^{13}C NMR spectra of pinacol.

[^{1}H, δ in ppm vs TMS, 400 MHz]: 2.65 (d, C); 1.13 (d, B)

[^{13}C, δ in ppm vs TMS, 100 MHz]: 75.3 (A); 25.2 (B)

1.3. EPR Spectroscopy

The formation of the ketyl-type radical dimerization product **D** was established by NMR spectroscopy. To illustrate the presence of the corresponding aroyl-type radical recombination product **B**, we recorded EPR spectra of 50 mM **1** in acetonitrile under light-irradiation in the presence/absence of triethylamine (**TEA.** 100 mM). Here, electron transfer from **TEA** to **B** leads to the formation of the benzil radical anion **B**$^{\bullet-}$ (Figure S**8**), which in turn can act as a reducing agent (Scheme 2, main text). The EPR resonance of **B**$^{\bullet-}$ is not observed in absence of **TEA**.[1]

Cw-EPR spectra were recorded on a Bruker X-band spectrometer (EMX 100 kHz field modulation) at room temperature with 0.025 mT field modulation amplitude. The signals correspond to the steady-state concentration of radicals accumulated in a flow system (0.4 mm quartz flat cell) under continuous irradiation. Simulation were performed using the WinSim 2002 software.[2] Continuous irradiation was conducted using a Hamamatsu 10 Lightingcure LC4 (Hg-Xe lamp, 3500 mW/cm^2, λ_{max}=365 nm).

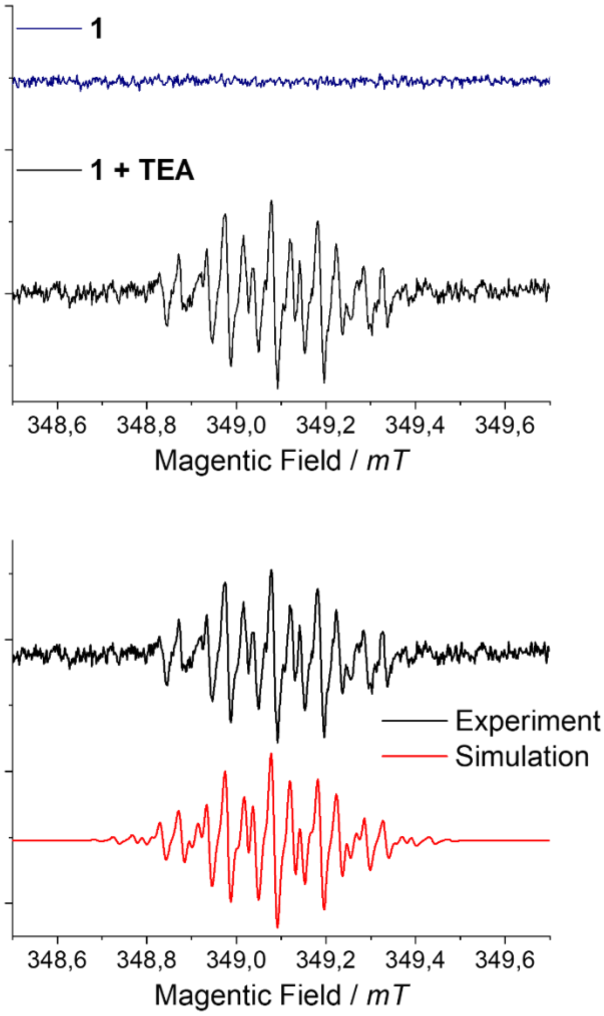

Figure S8. Top: EPR spectra of solutions containing either **1** (blue) or **1** and **TEA** (black). Both spectra were recorded under continuous light irradiation. Bottom: Corresponding simulation (red).

2. Kinetic Modelling

Kinetic modelling can be utilized to fit and validate experimental data, as well as to make predictions regarding the feasibility of photo-induced metal nanoparticle synthesis. This kinetic modelling is conducted using COPASI, an open-source software [https://doi.org/10.1093/bioinformatics/btl485]. We present exemplary models for the reduction of metallic nanoparticles by photo-initiators (for both models, the original COPASI files are available).

Scheme S1 summarizes the reduction of divalent metal ions (**M**$^{2+}$) to metallic nanoparticles (**MNP**) upon irradiation of a α-hydroxyketone photoinitiator: We consider the following reactions in the model: Reaction (1) describes the primary α-cleavage of a α-hydroxyketone upon irradiation and intersystem crossing, producing benzoyl radical **A•** and ketyl radical **K•** comprising quantum yield $\Phi_{Photolysis}$. **K•** transfers one electron to **M**$^{2+}$ (or **M**$^{+}$), yielding acetone (Reactions (2) and (3)). Importantly, the agglomeration of metal atoms **M** towards nanoparticles must be included here. An additional source of **M**^{0} is the disproportionation of **M**$^{+}$ (Reaction (5)). The presence of **MNP** accelerates the nucleation process (Reaction (6)).[3]

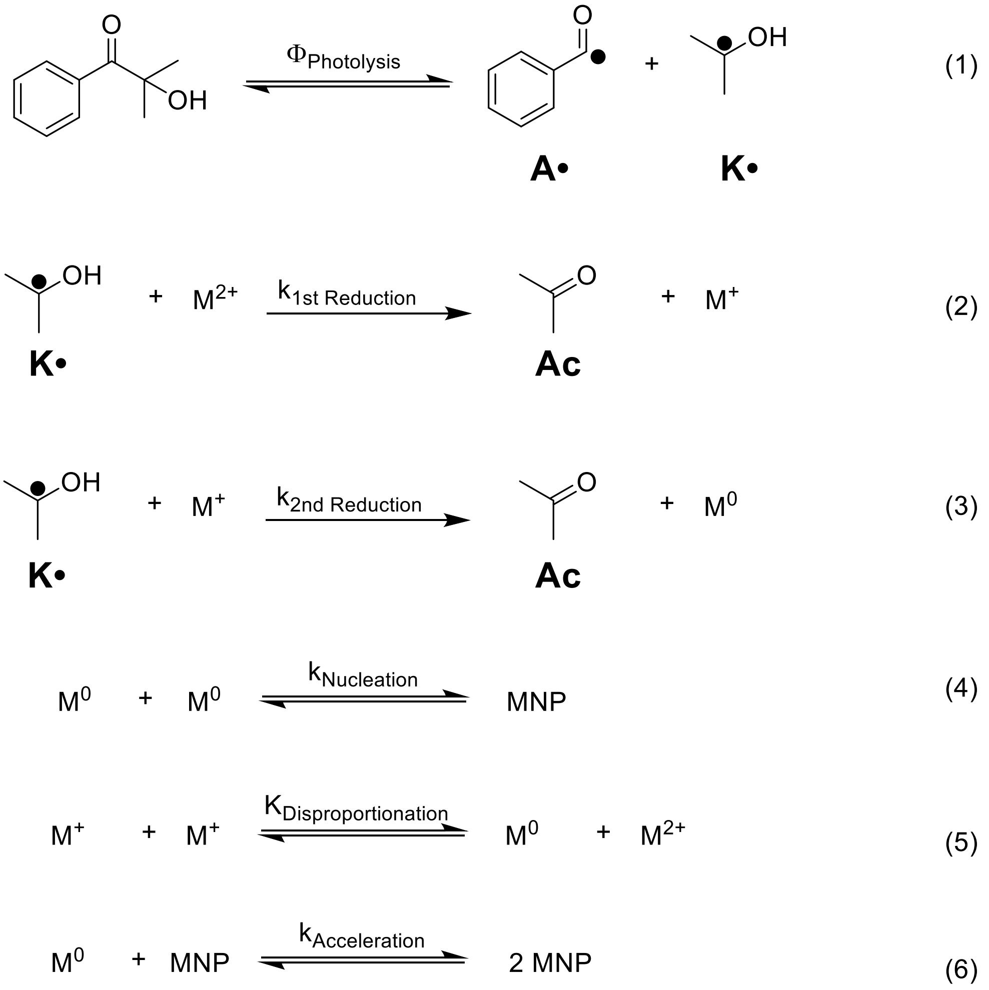

Scheme S1. Reaction system used for kinetic modelling of the reduction of divalent metal ions (**M**$^{2+}$) to metallic nanoparticles (**MNP**) upon irradiation of a α-hydroxyketone photoinitiator.

Scheme S2 summarizes the reduction of divalent metal ions (**M^{2+}**) to metallic nanoparticles (**MNP**) upon irradiation of a **BAPO** photoinitiator: Here, in addition to the reactions shown in Scheme S1, we additionally consider that in the presence of R–OH (R = H or alkyl), the primary phosphanoyl radical **P•** undergoes hydrolysis to give the persistent radical **C•** (Reaction (2)). In our model, **C•** transfers one electron to **M^{2+}** (or **M^{+}**), yielding a mono(acyl)phosphine oxide **MAPO** (Reactions (4) and (5)). Alternatively, the phosphanoyl radical can be oxidized to cation **P^{+}** (Reaction (3)).[4]

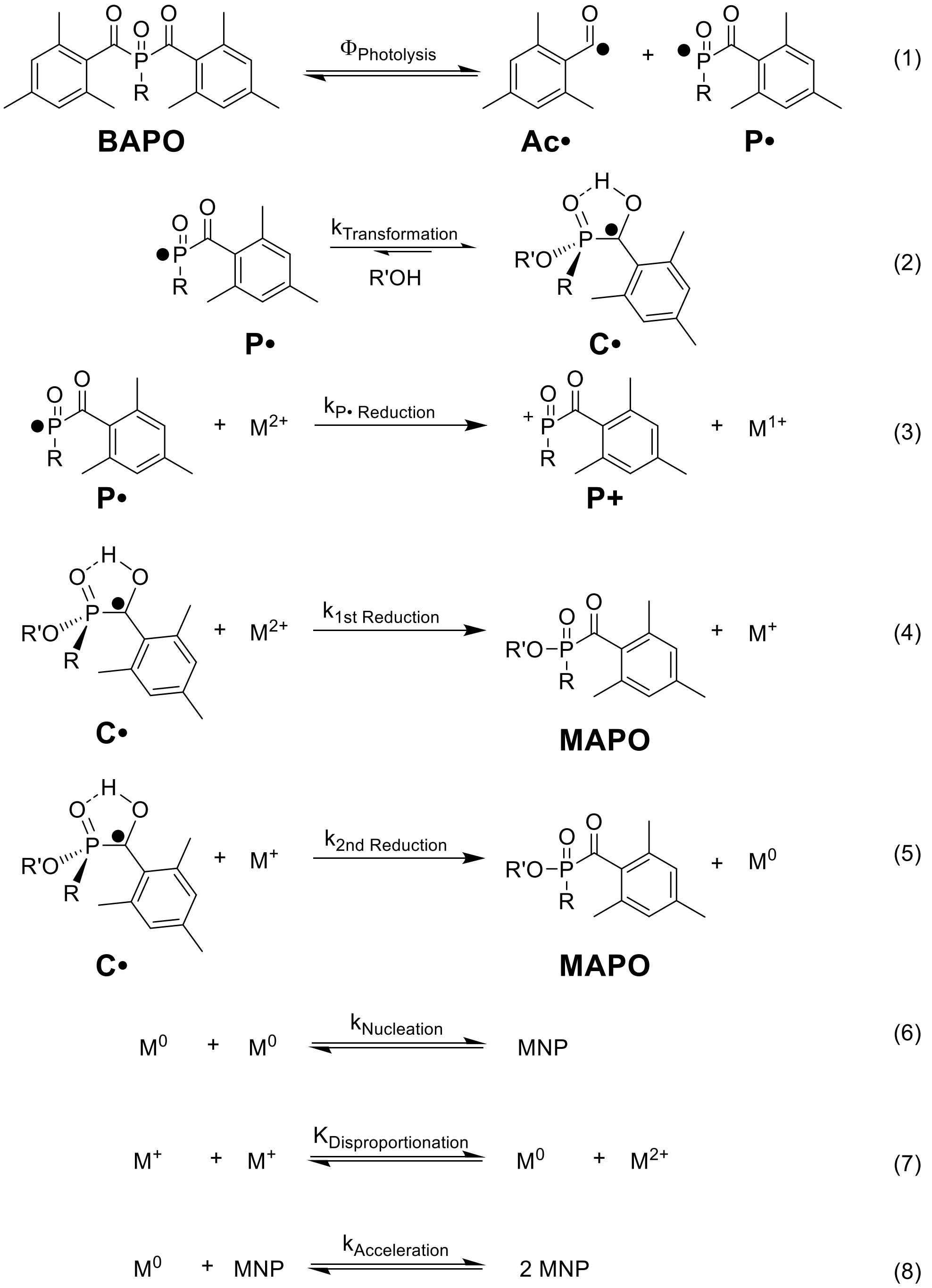

Scheme S2. Reaction system used for kinetic modelling of the reduction of divalent metal ions (**M^{2+}**) to metallic nanoparticles (**MNP**) upon irradiation of a **BAPO** photo-initiator in alcoholic or aqueous media.

3. References

[1] M. Schmallegger, G. Gescheidt, *Monatsh. Chem.* **2018**, *149*, 499–504.

[2] D.R. Duling, *J. Magn. Reson.* **1994**, *104*, 105-110.

[3] N. L. Pacioni, A. Pardoe, K. L. McGilvray, M. N. Chrétien, J. C. Scaiano, *Photochem. Photobiol. Sci.* **2010**, *9*, 766–774.

[4] M. Schmallegger, H. Grützmacher, G. Gescheidt, *Chemphotochem* **2022**, *6*.
